# Supplementary material for: FOXP3 recognizes microsatellites and bridges DNA through multimerization
Source: Nature. 2023 Nov 29;624(7991):433–41. doi: 10.1038/s41586-023-06793-z (PMC10719092; doi:10.1038/s41586-023-06793-z)
Supplement: Supplementary file 1 — Supplementary Fig. 1 (gating strategies for FACS analysis) and Supplementary fig. 2 (uncropped full scans for all blot results). [file 41586_2023_6793_MOESM1_ESM.pdf]

nature portfolio

https://doi.org/10.1038/s41586-023-06793-z

# Supplementary information

# FOXP3 recognizes microsatellites and bridges DNA through multimerization

In the format provided by the authors and unedited

# Supplementary Figure 1

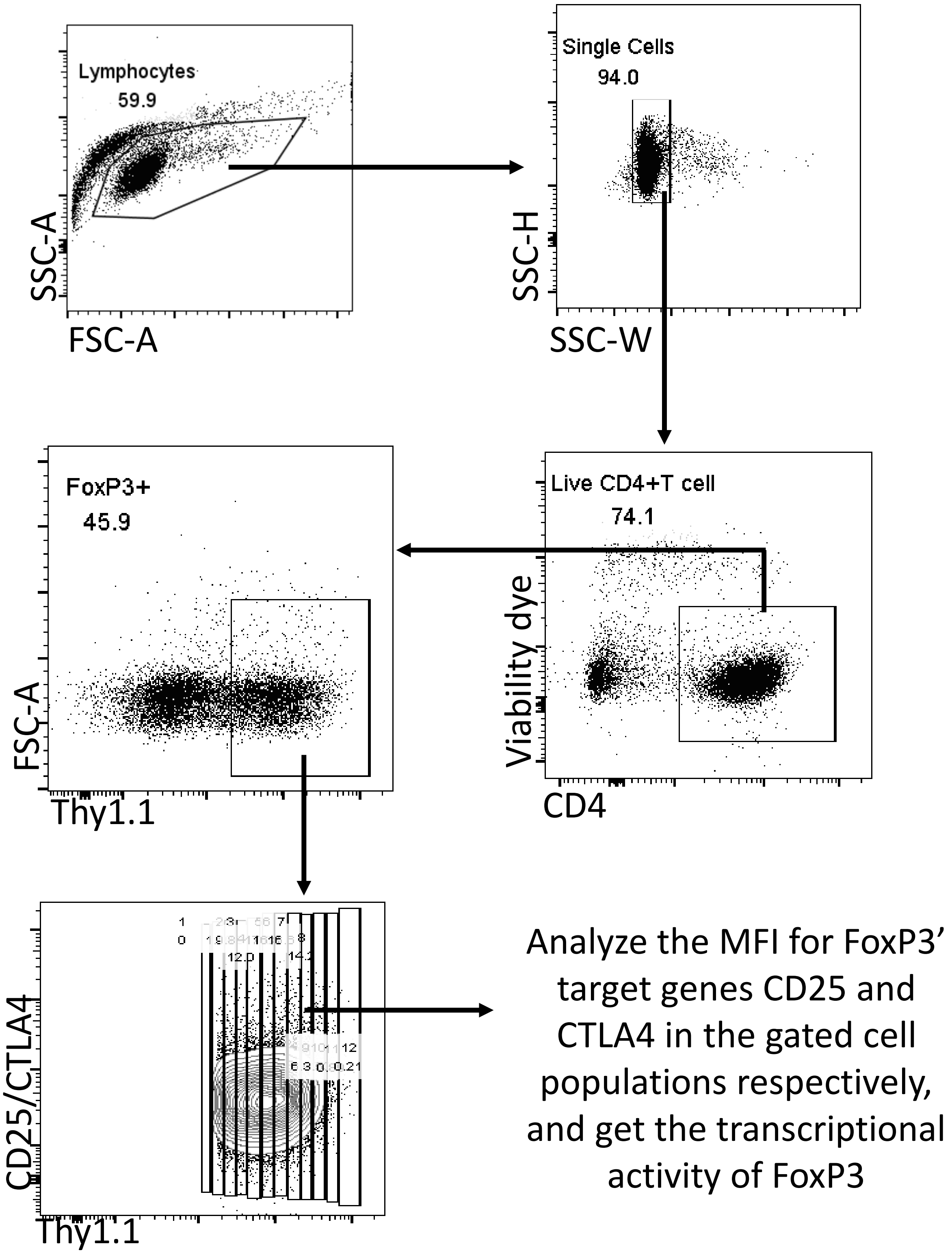


**Gating strategies for FACS analysis**

# Supplementary Figure 2

## For Figure 1

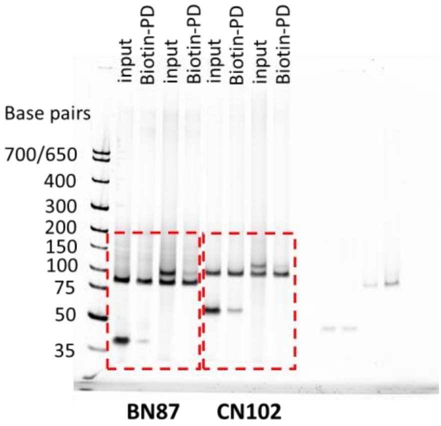


Used in Figure 1c. Right panel.

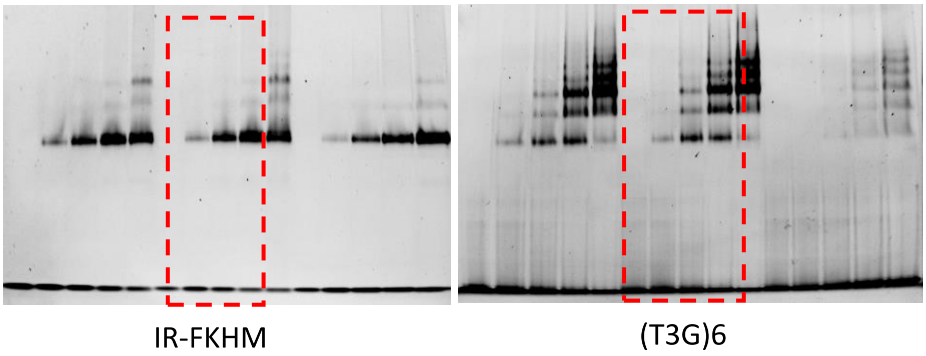


Used in Figure 1i

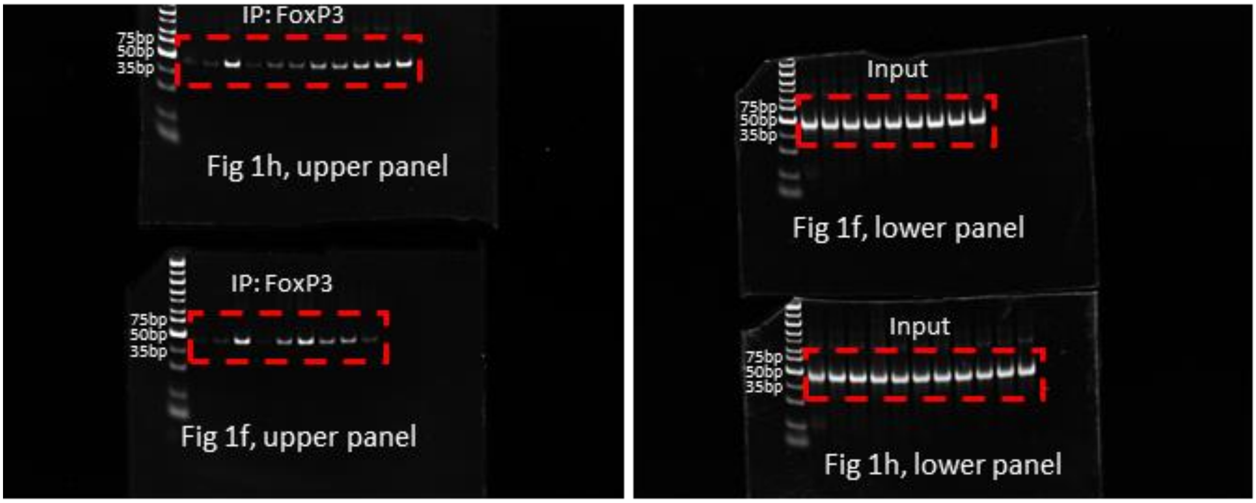


Used in Figure 1f and 1h

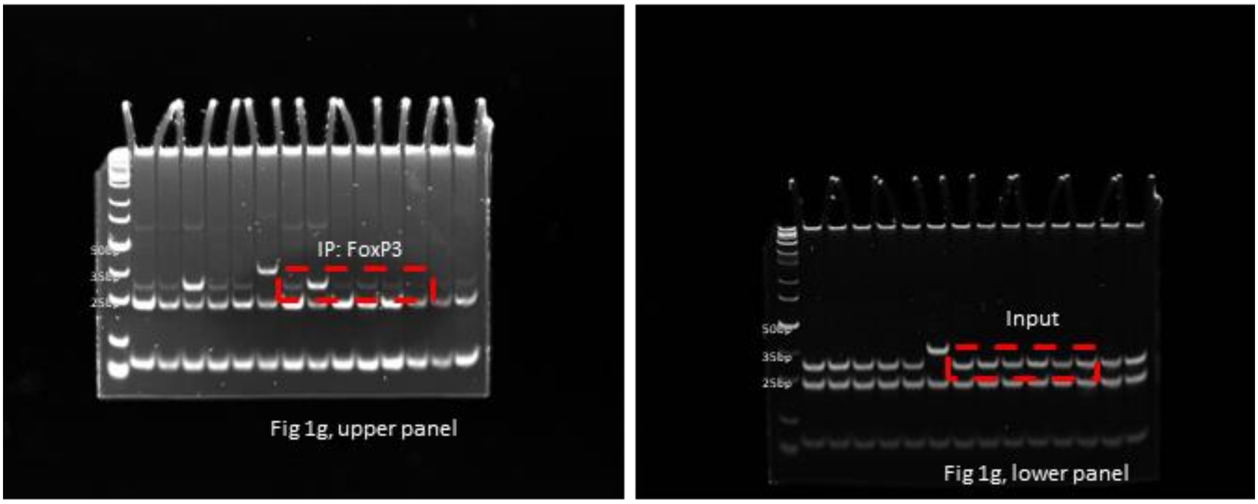


Used in Figure 1g

**For Figure 2 and Figure 3**

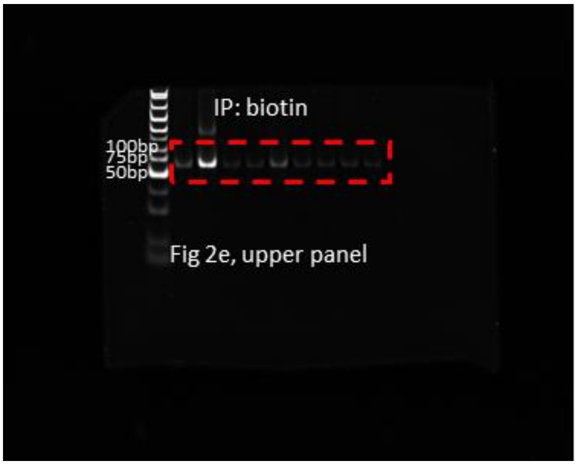


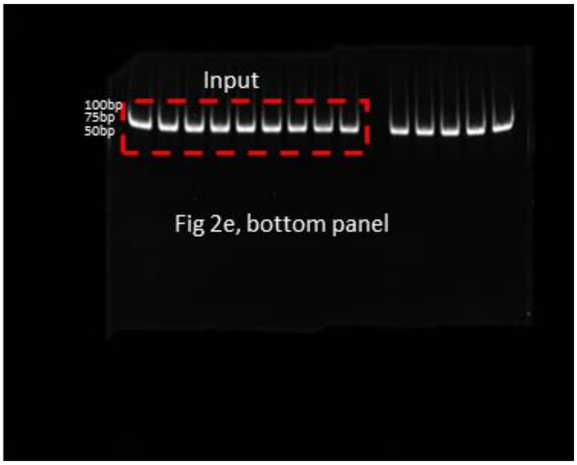


Used in Figure 2e

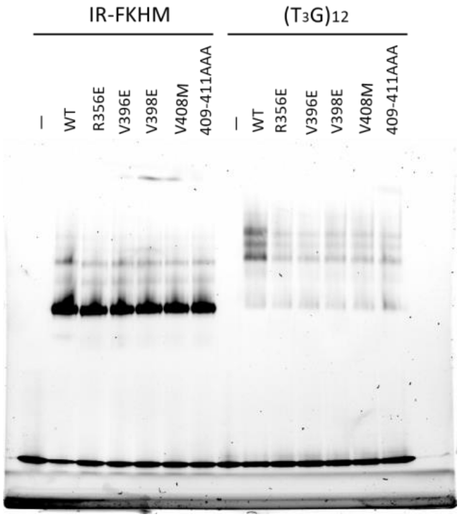


Used in Figure 3b

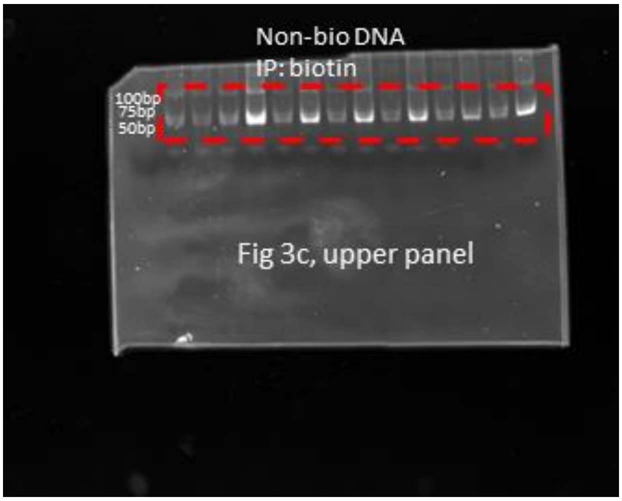


Used in Figure 3c, upper panel

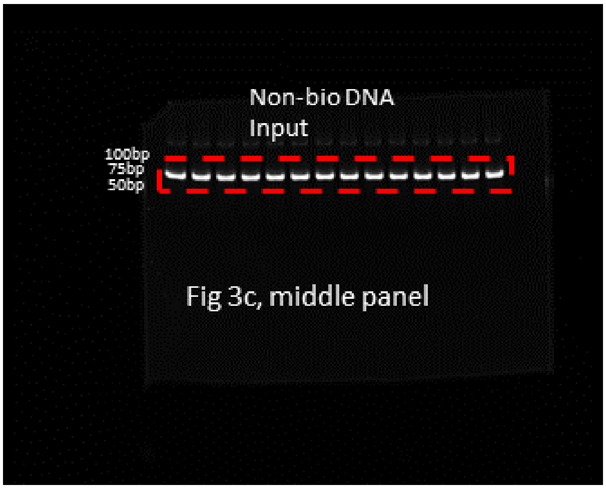


Used in Figure 3c, middle panel

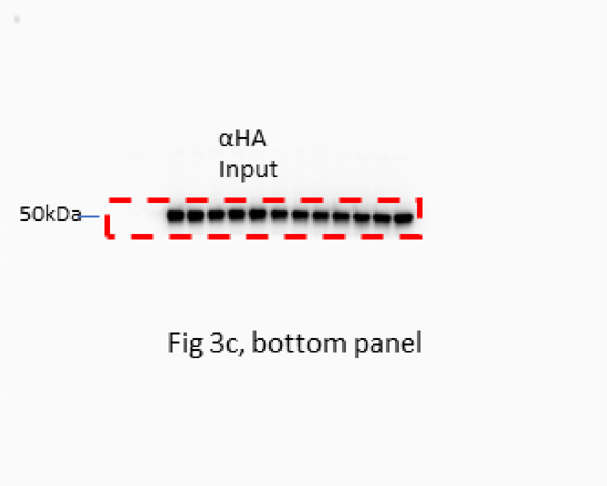


Used in Figure 3c, bottom panel

## For Figure 4

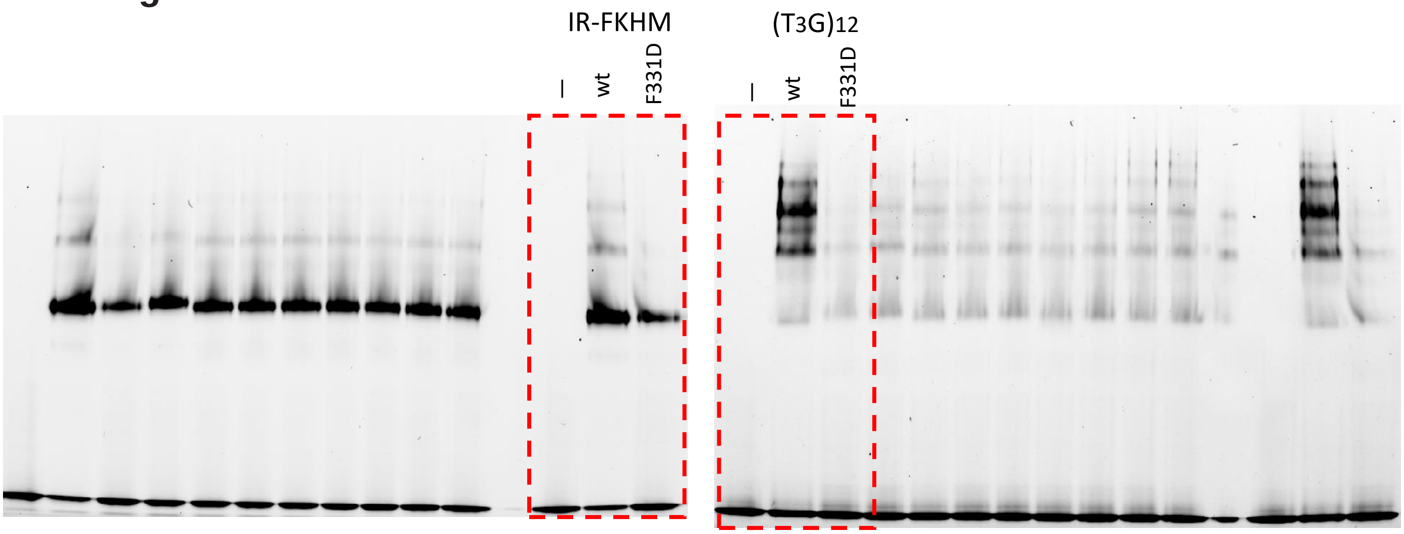


Used in Figure 4b

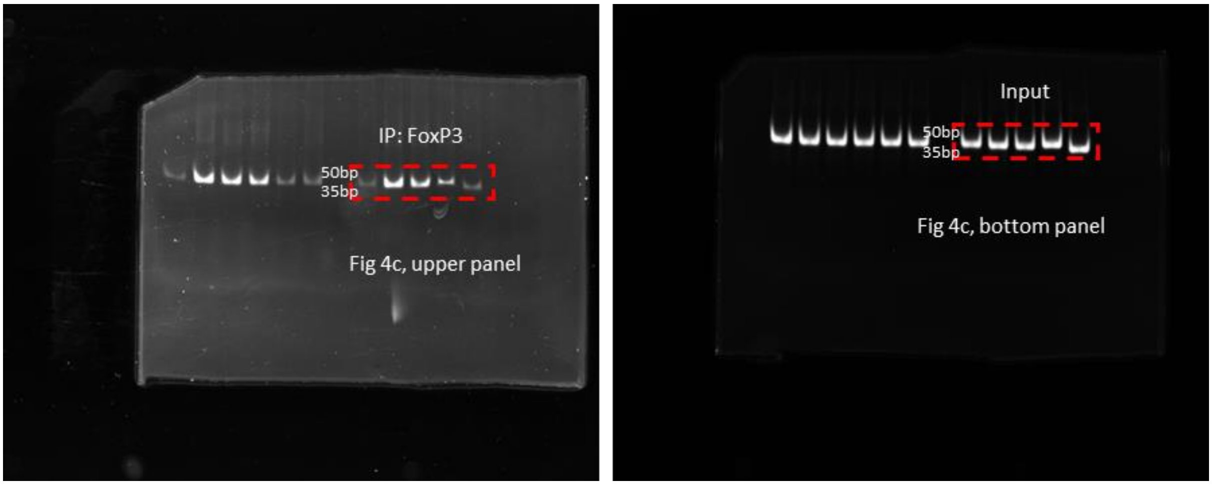


Used in Figure 4c

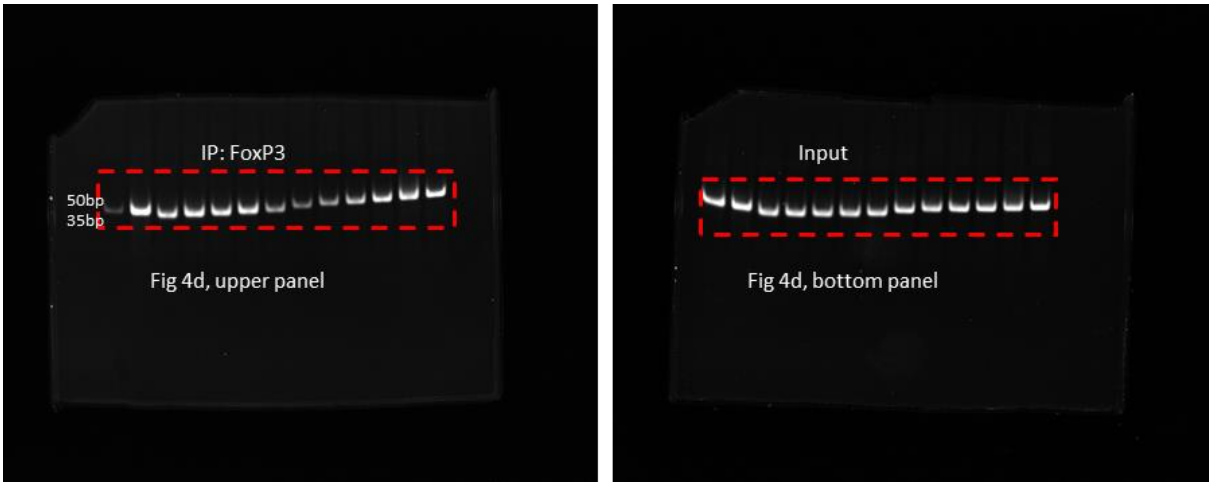


Used in Figure 4d

# For Figure 4

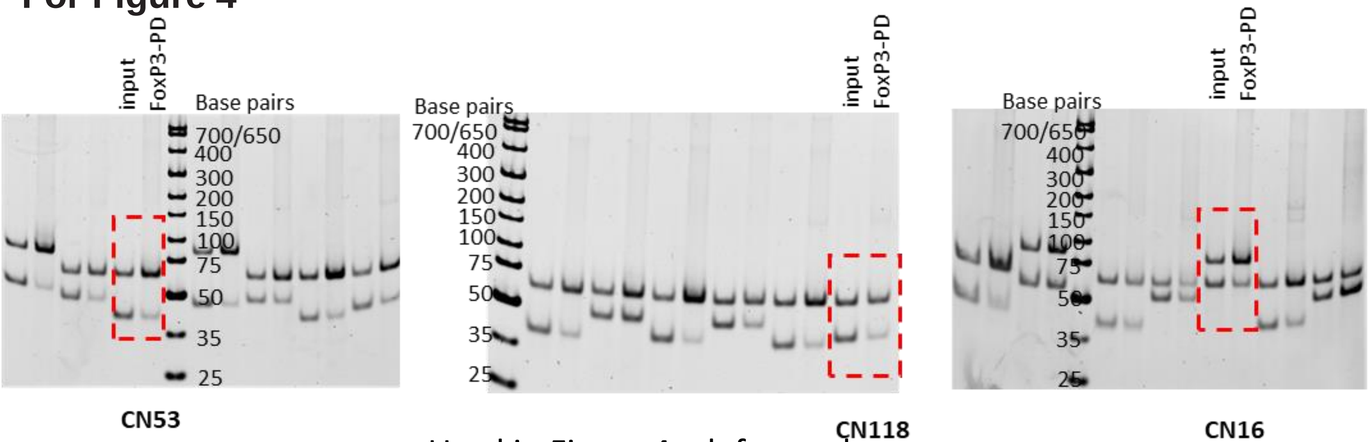


Used in Figure 4e, left panel

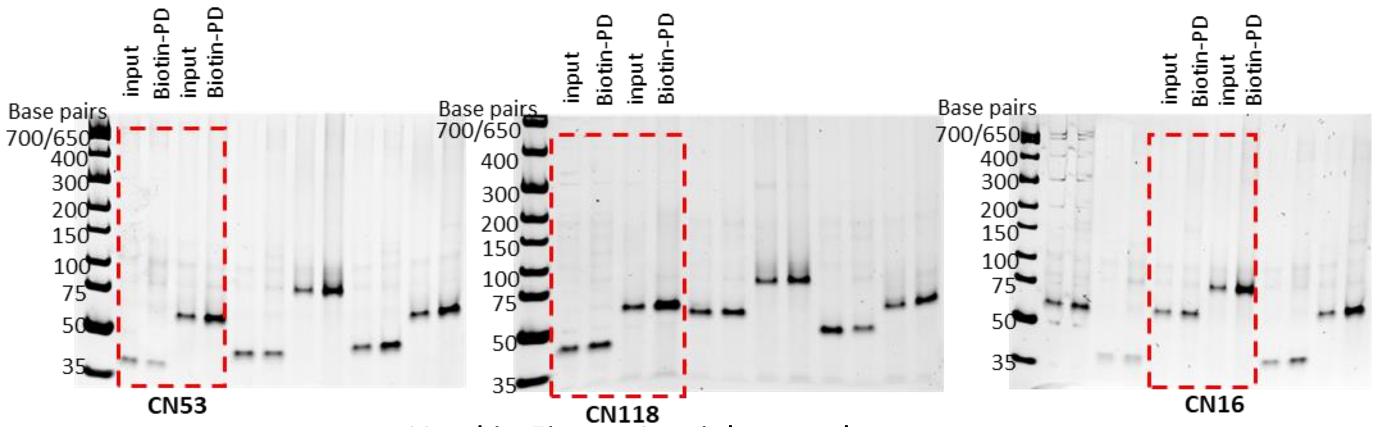


Used in Figure 4e, right panel

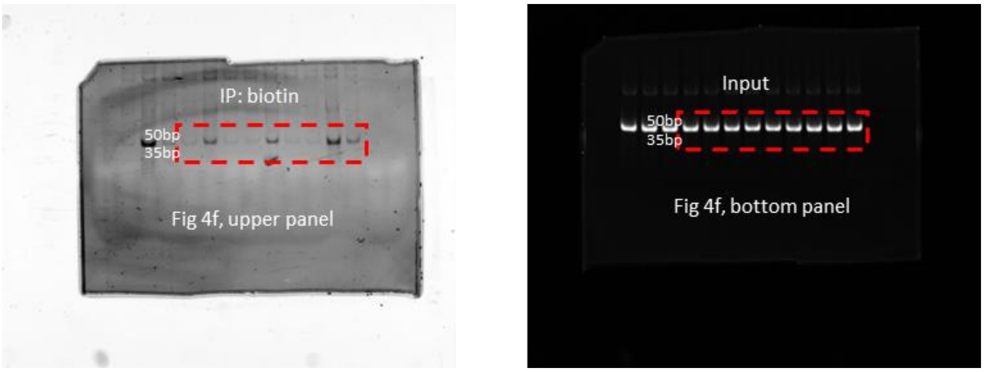


Used in Figure 4f

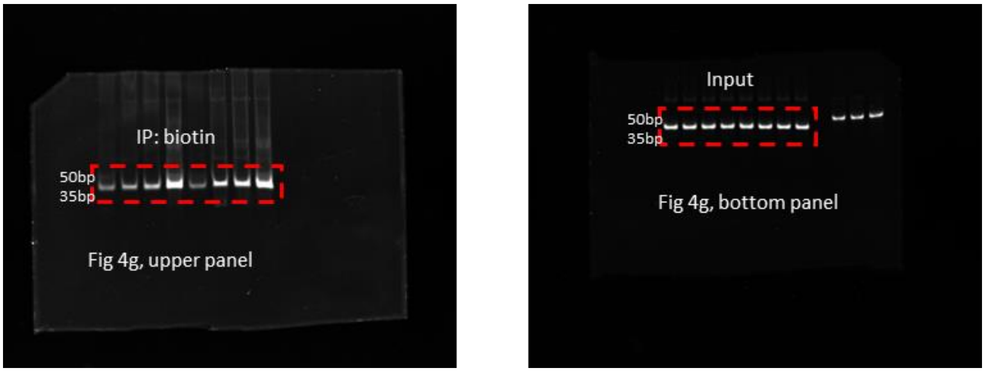


Used in Figure 4g

## For Figure 5 and ED Figure 1

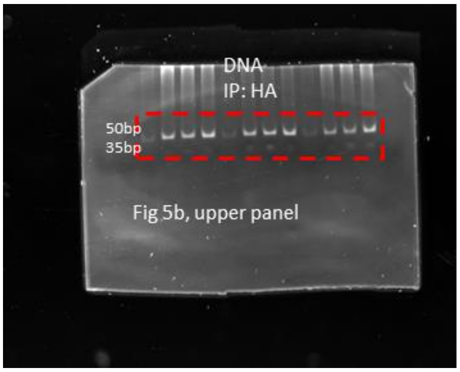


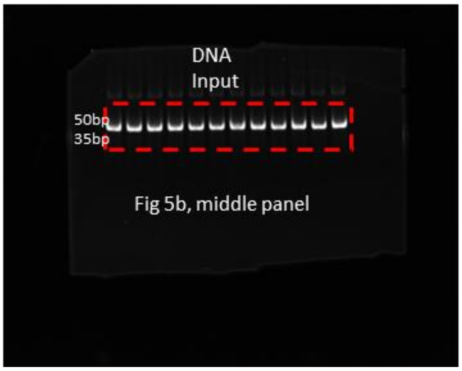


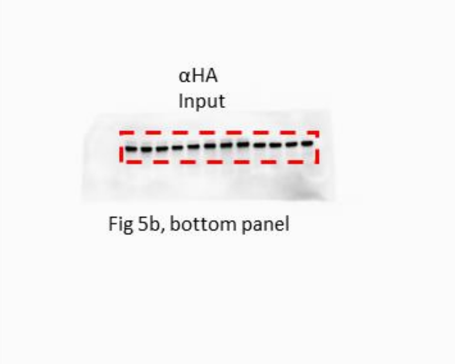


Used in Figure 5b

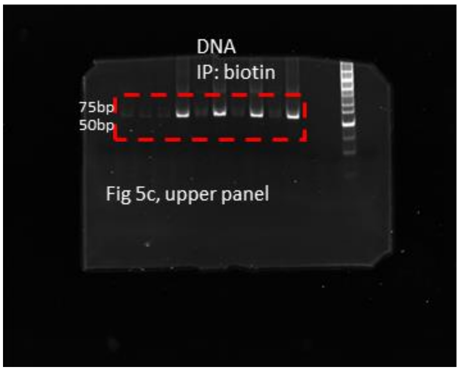


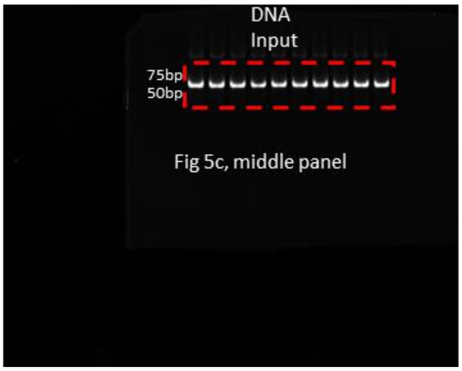


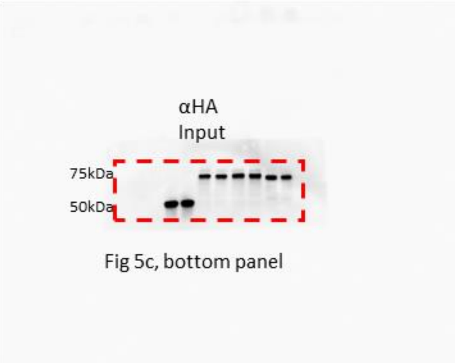


Used in Figure 5c

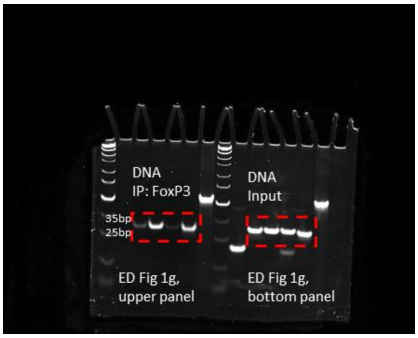


Used in ED Figure 1g

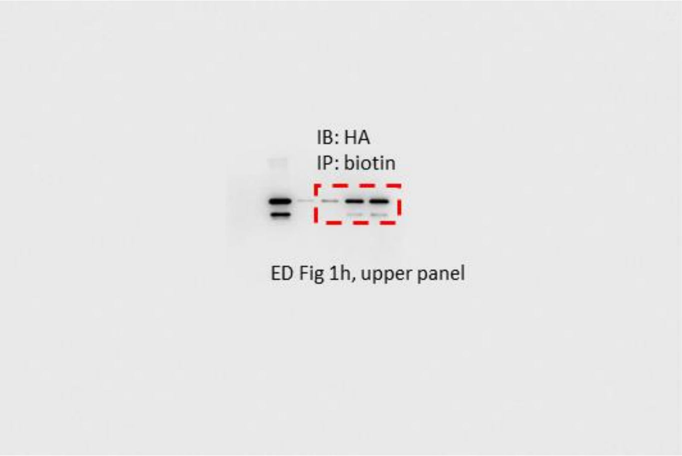


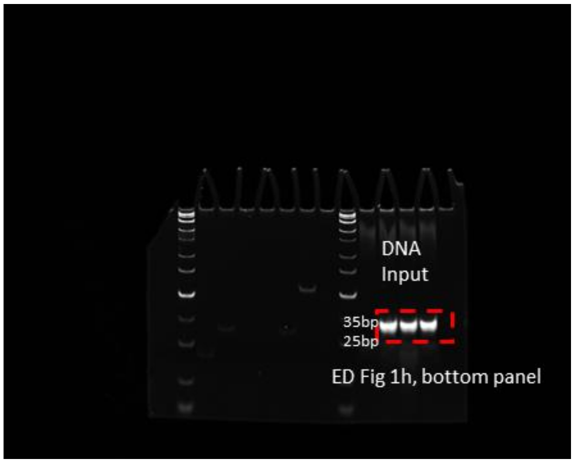


Used in ED Figure 1h

**For ED Figure 1**

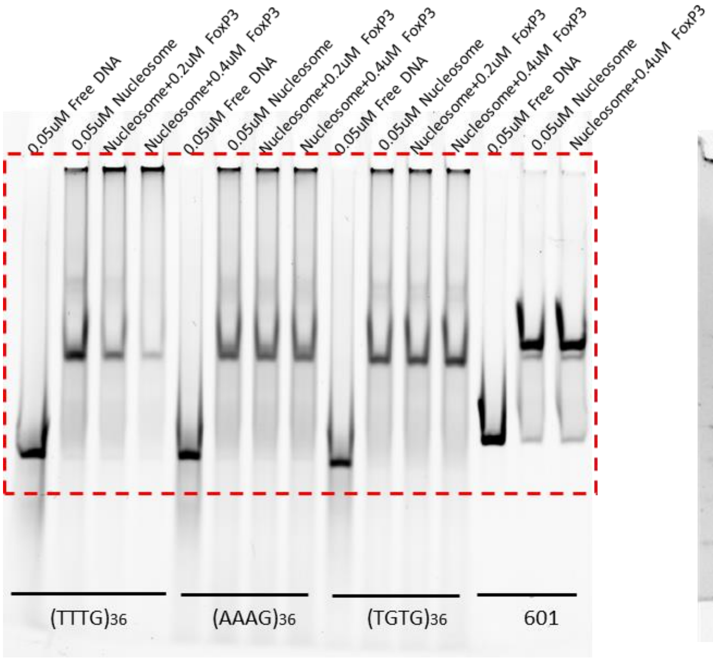


Used in ED Figure 1i

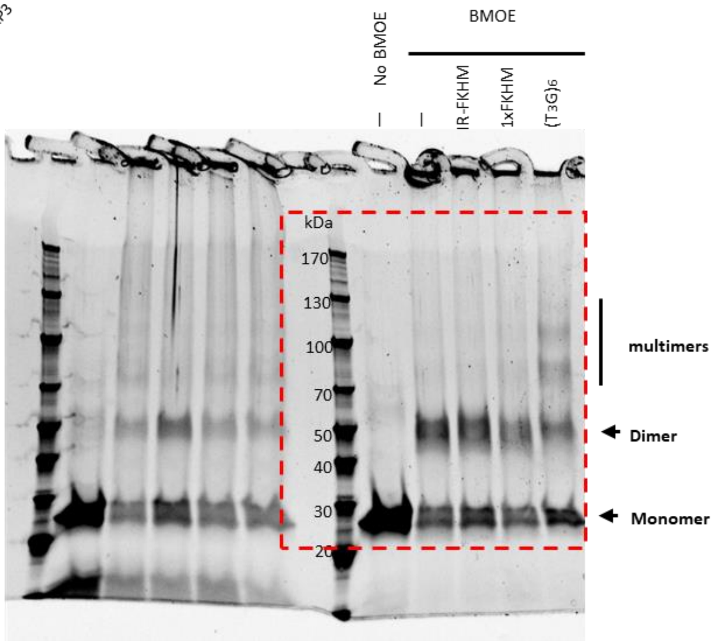


Used in ED Figure 1j

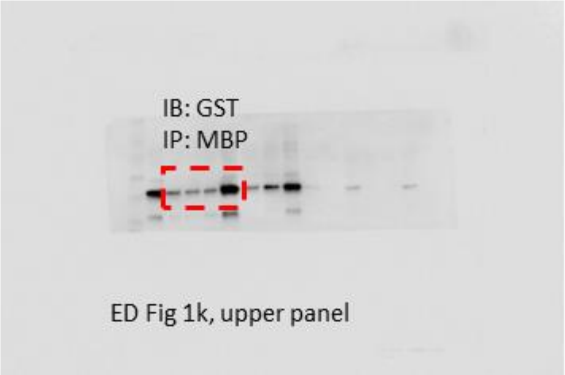


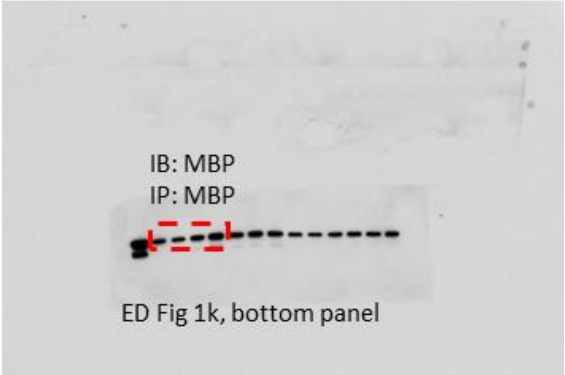


Used in ED Figure 1k

## For ED Figure 2 and 3

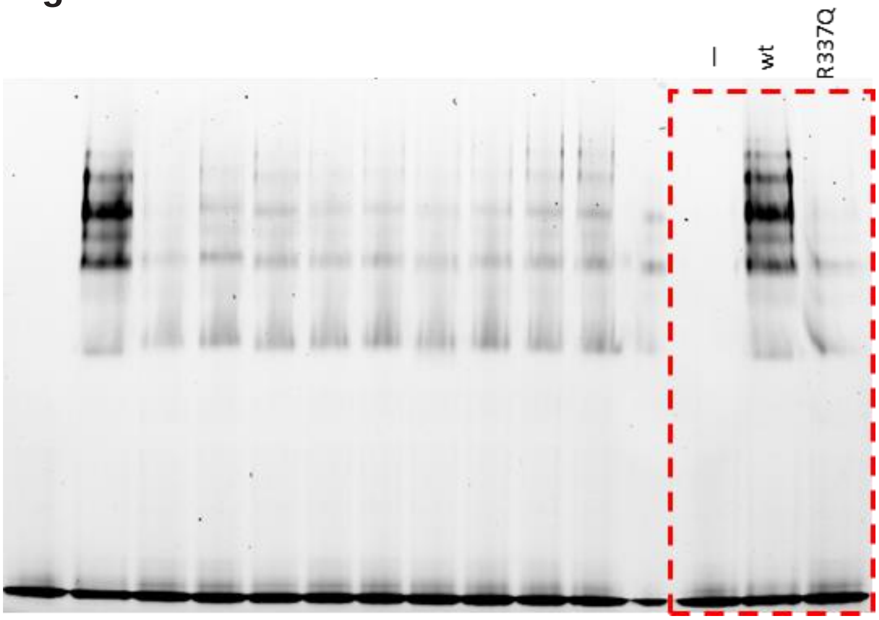


Used in ED Figure 2h

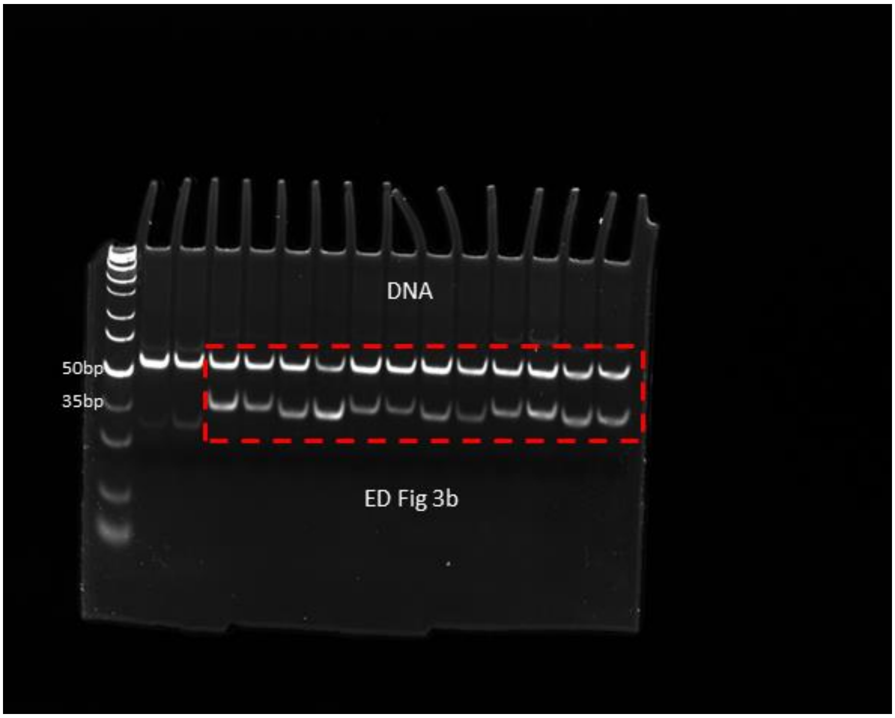


Used in ED Figure 3b

## For ED Figure 3

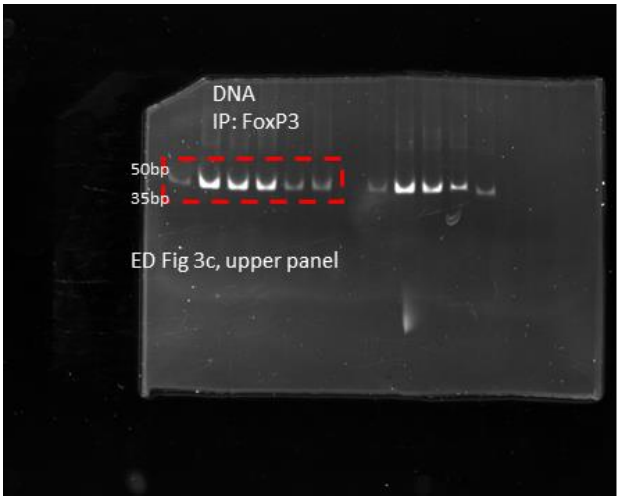


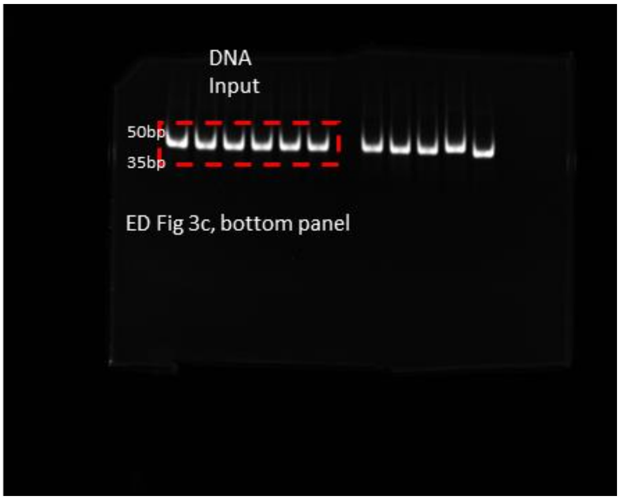


Used in ED Figure 3c

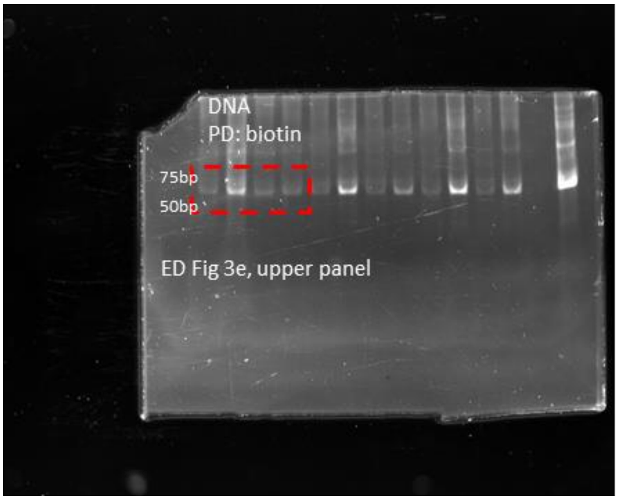


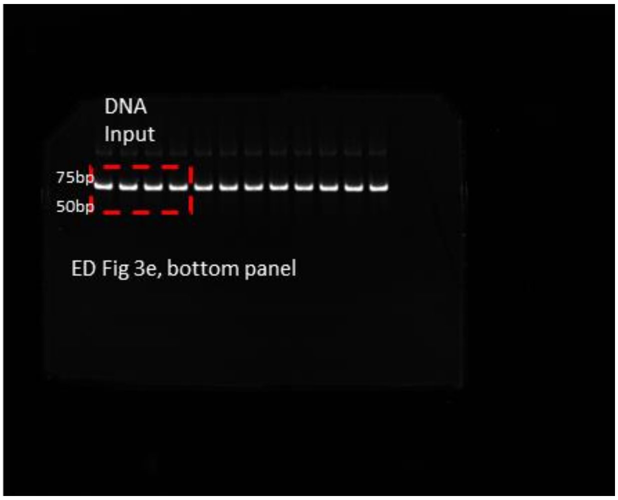


Used in ED Figure 3e

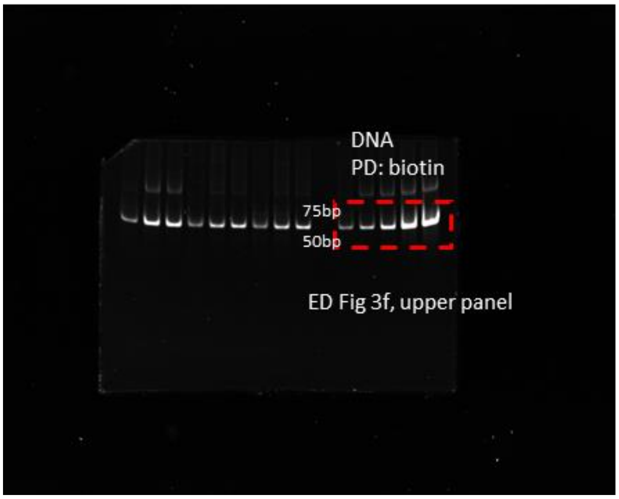


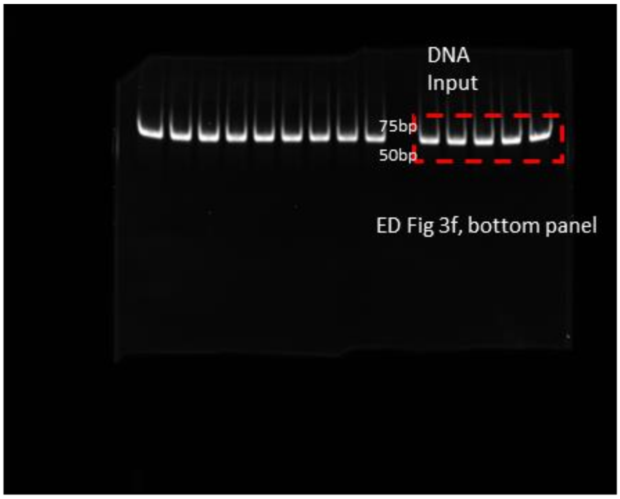


Used in ED Figure 3f

## For ED Figure 5

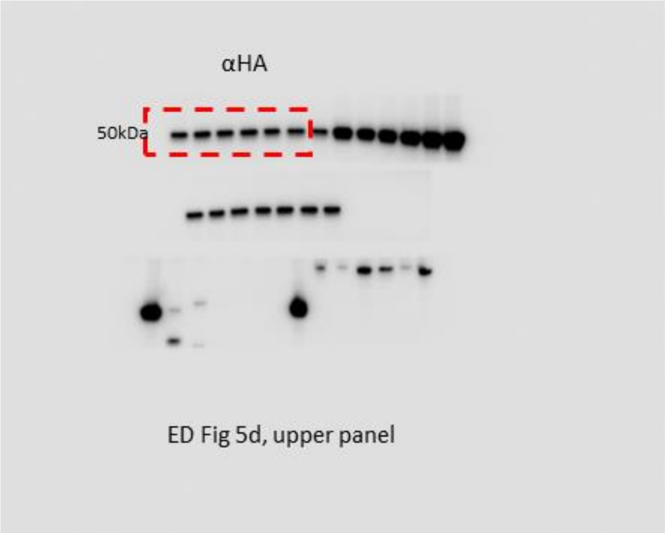


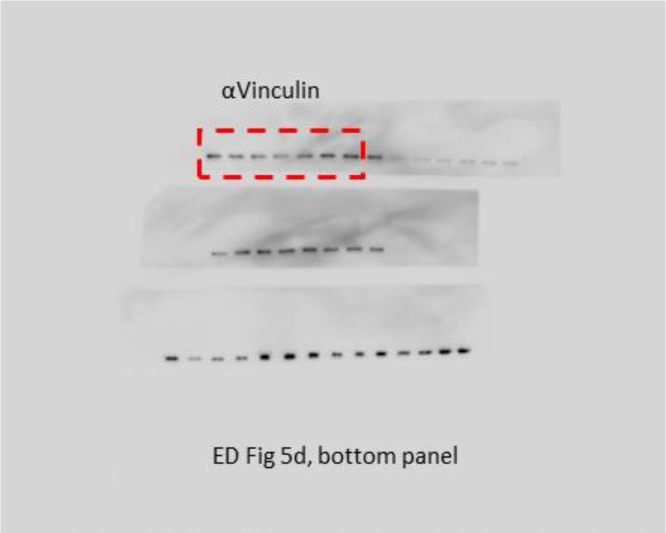


Used in ED Figure 5d

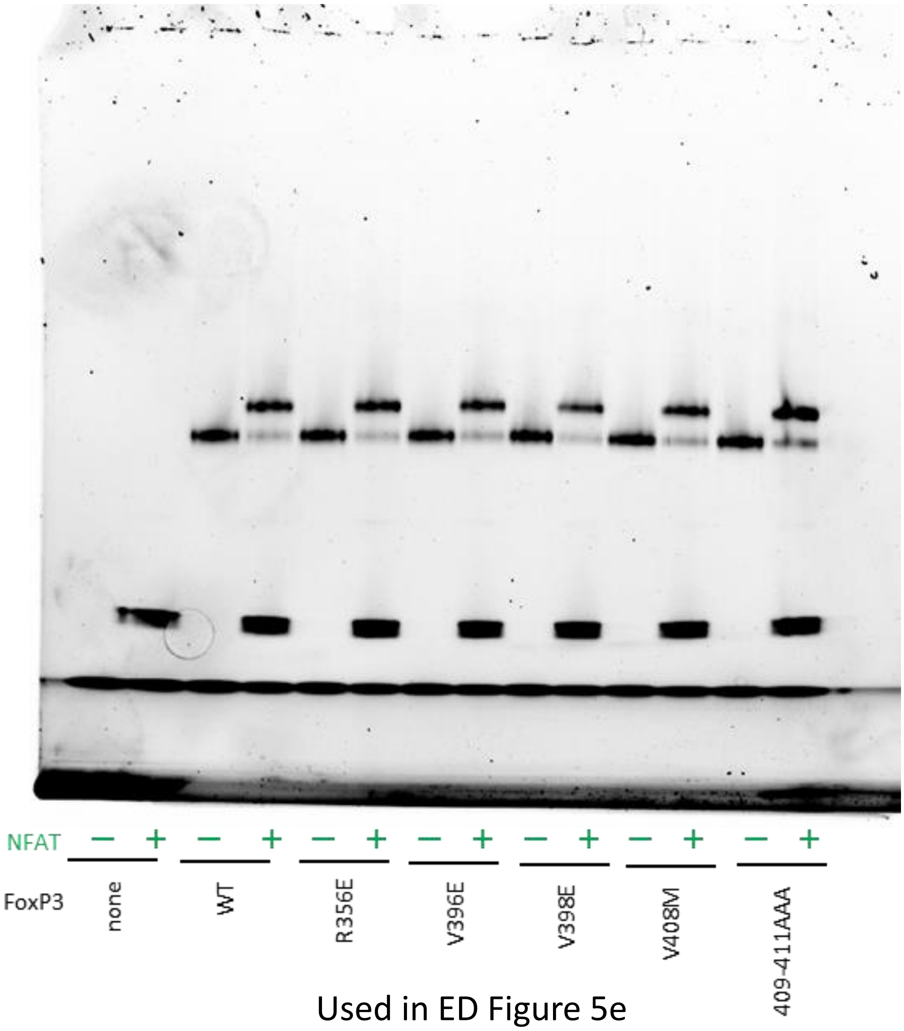


Used in ED Figure 5e

# For ED Figure 6

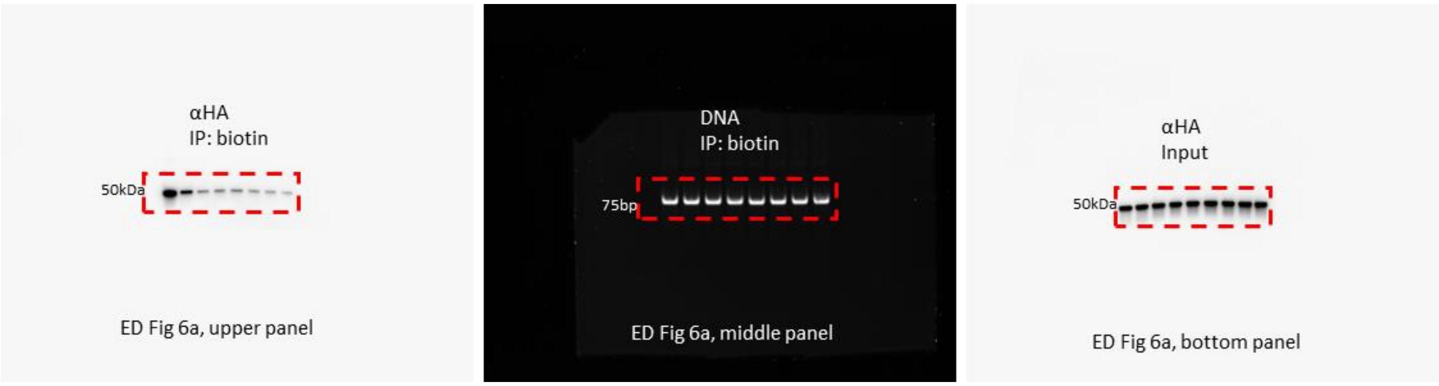


Used in ED Figure 6a

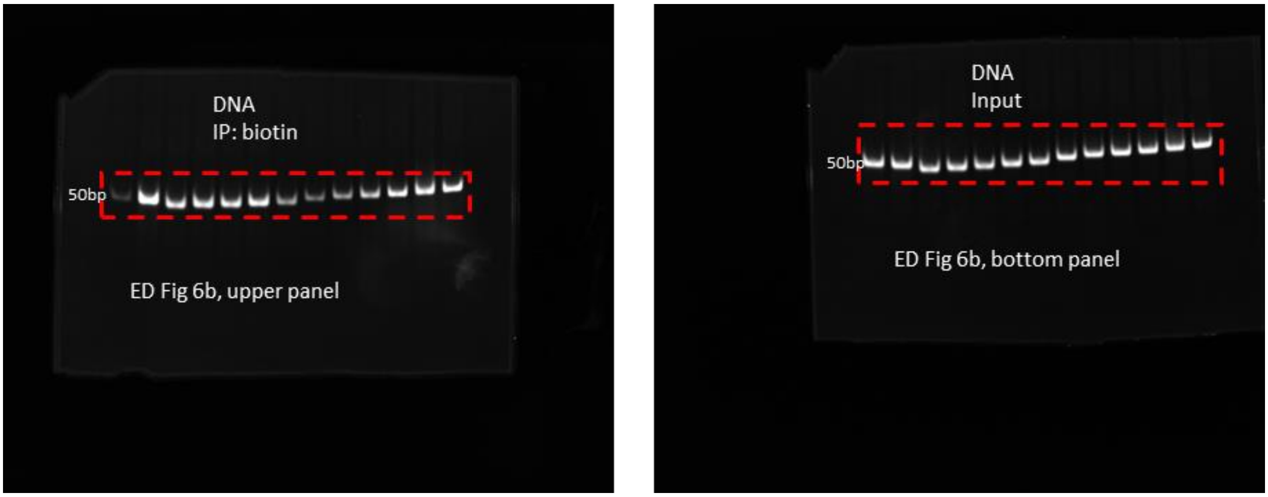


Used in ED Figure 6b

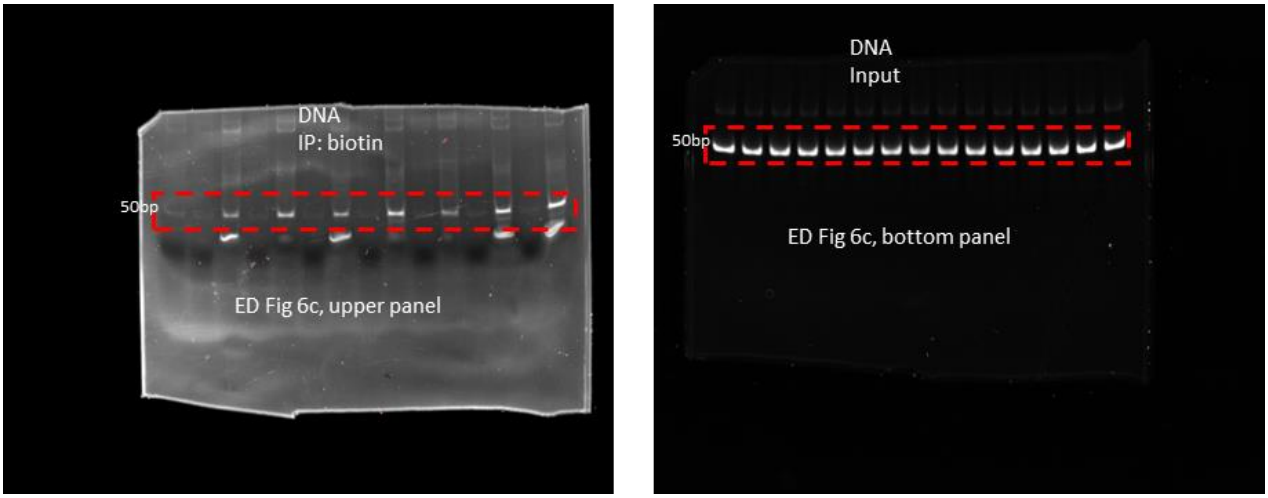


Used in ED Figure 6c

# For ED Figure 6

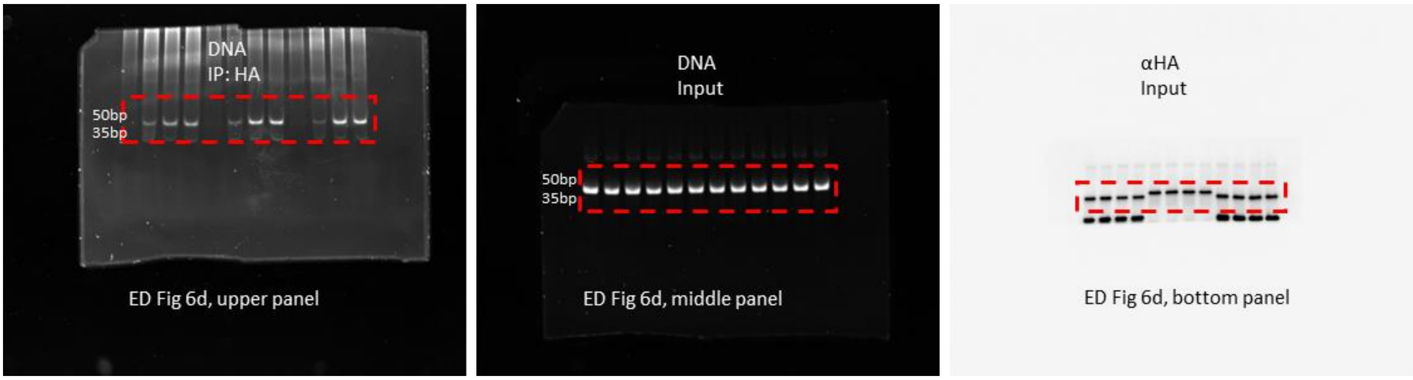


Used in ED Figure 6d

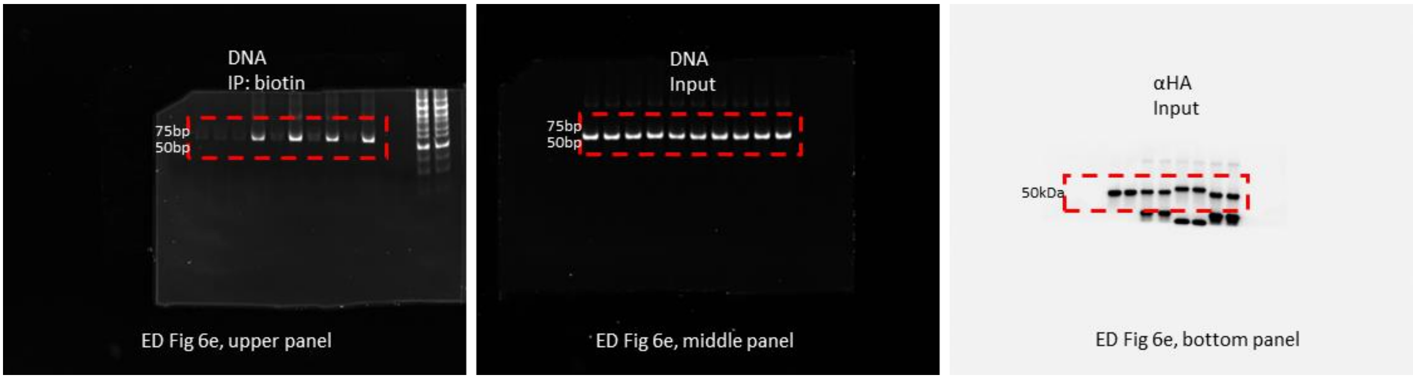


Used in ED Figure 6e

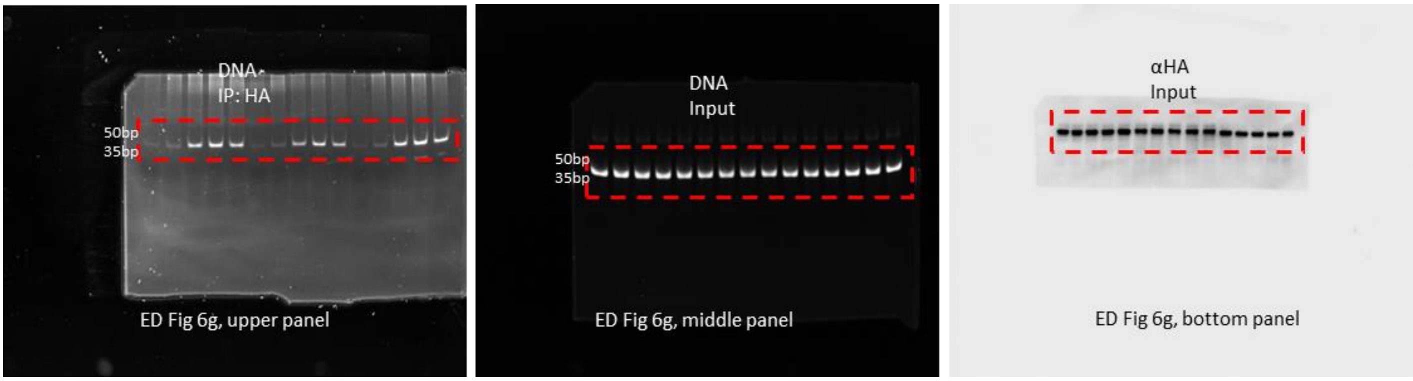


Used in ED Figure 6g

# Supplementary Tables

**Supplementary Table 1.** Summary of FoxP3 binding motifs. 1a, FoxP3 binding motif *in vitro*. 1b, FoxP3 binding motifs *in vivo*. 1c, other FKH TFs' binding motifs.

**Supplementary Table 2.** List of DNA oligos used in this study. 2a, allelic bias sites information. 2b, DNA oligo sequences.

**Supplementary Table 3.** Chromatin contacts at FoxP3-bound anchors. 3a, summary of the contacts with frequency>5 in WT Treg HiC and connected by two TnG anchors. 3b-3f, with an increasing FoxP3 PLAC-seq count threshold (>5, 10, 25, 50, 75 respectively), all the anchor information is displayed. 3g, genomic annotation for the CNR peaks overlapping with PLAC-seq counts above the threshold of 75.
