# Supplementary material for: FOXP3 recognizes microsatellites and bridges DNA through multimerization
Source: Nature. 2023 Nov 29;624(7991):433–41. doi: 10.1038/s41586-023-06793-z (PMC10719092; doi:10.1038/s41586-023-06793-z)
Supplement: Supplementary file 2 — Reporting Summary [file 41586_2023_6793_MOESM2_ESM.pdf]

nature portfolio

Corresponding author(s): Sun Hur

Last updated by author(s): Oct 11, 2023

Reporting Summary

Nature Portfolio wishes to improve the reproducibility of the work that we publish. This form provides structure for consistency and transparency in reporting. For further information on Nature Portfolio policies, see our Editorial Policies and the Editorial Policy Checklist.

Statistics

For all statistical analyses, confirm that the following items are present in the figure legend, table legend, main text, or Methods section.

n/a	Confirmed	
☐	☒	The exact sample size (*n*) for each experimental group/condition, given as a discrete number and unit of measurement
☐	☒	A statement on whether measurements were taken from distinct samples or whether the same sample was measured repeatedly
☐	☒	The statistical test(s) used AND whether they are one- or two-sided<br>*Only common tests should be described solely by name; describe more complex techniques in the Methods section.*
☒	☐	A description of all covariates tested
☒	☐	A description of any assumptions or corrections, such as tests of normality and adjustment for multiple comparisons
☐	☒	A full description of the statistical parameters including central tendency (e.g. means) or other basic estimates (e.g. regression coefficient) AND variation (e.g. standard deviation) or associated estimates of uncertainty (e.g. confidence intervals)
☐	☒	For null hypothesis testing, the test statistic (e.g. *F*, *t*, *r*) with confidence intervals, effect sizes, degrees of freedom and *P* value noted<br>*Give P values as exact values whenever suitable.*
☒	☐	For Bayesian analysis, information on the choice of priors and Markov chain Monte Carlo settings
☒	☐	For hierarchical and complex designs, identification of the appropriate level for tests and full reporting of outcomes
☒	☐	Estimates of effect sizes (e.g. Cohen's *d*, Pearson's *r*), indicating how they were calculated

Our web collection on statistics for biologists contains articles on many of the points above.

Software and code

Policy information about availability of computer code

Data collection	FACS data was collected by BD FACSDiva™ Software v. 6.1.3
Data analysis	Softwares and versions:<br>Trimmomatic (version 0.36); Bowtie2 (version 2.3.4.3); Macs2 (version 2.1.1.20160309); DiffBind (version 4.3); Bedtools (version 2.30.0); Meme Fimo (version 5.3.3); Samtools (version 1.9); Deeptools (version 3.0.2)

For manuscripts utilizing custom algorithms or software that are central to the research but not yet described in published literature, software must be made available to editors and reviewers. We strongly encourage code deposition in a community repository (e.g. GitHub). See the Nature Portfolio guidelines for submitting code & software for further information.

Data

Policy information about availability of data

All manuscripts must include a data availability statement. This statement should provide the following information, where applicable:

- Accession codes, unique identifiers, or web links for publicly available datasets
- A description of any restrictions on data availability
- For clinical datasets or third party data, please ensure that the statement adheres to our policy

Naked genomic DNA pulldown-seq, nucleosome pulldown-seq, FoxP3 mRNA-seq and FoxP3 ChIP-seq data have been deposited to the GEO database with the accession code of GSE243606. The structures and cryo-EM maps have been deposited to the PDB and the EMDB under the accession codes of 8SRP and EMD-40737

for the decameric FoxP3 in complex with DNA, and 8SRO and EMD-40736 for the central FoxP3 tetramer in complex with DNA (focused refinement). Other research materials reported here are available on request.

Research involving human participants, their data, or biological material

Policy information about studies with human participants or human data. See also policy information about sex, gender (identity/presentation), and sexual orientation and race, ethnicity and racism.

Reporting on sex and gender	N/A
Reporting on race, ethnicity, or other socially relevant groupings	N/A
Population characteristics	N/A
Recruitment	N/A
Ethics oversight	N/A

Note that full information on the approval of the study protocol must also be provided in the manuscript.

Field-specific reporting

Please select the one below that is the best fit for your research. If you are not sure, read the appropriate sections before making your selection.

☒ Life sciences ☐ Behavioural & social sciences ☐ Ecological, evolutionary & environmental sciences

For a reference copy of the document with all sections, see nature.com/documents/nr-reporting-summary-flat.pdf

Life sciences study design

All studies must disclose on these points even when the disclosure is negative.

Sample size	For the FoxP3 transcriptional activity assay in CD4+ T cells, we utilized 0.2 million activated CD4+ T cells per well in a 96-well plate for each sample. For FoxP3 ChIP-seq analysis, we collected 5 million CD4+ T cells for each sample. For mRNA-seq analysis, we sorted and collected 1 million transduced CD4+ T cells, and then performed total RNA extraction for each sample.<br>The specific sample size for each experiment is described in individual figure legends. Each data point on the graphs represents an individual sample.
Data exclusions	None
Replication	All biochemical assays, pull-downs, bridging assays, FoxP3 transcriptional activity assay and T cell suppression assays were performed over 3 individual times. Only experimental data that were successfully replicated in all attempts are reported. See Figure legends for details.
Randomization	For immunofluorescence microscopy, images were taken at random locations on the cover slip. For negative-stain electron microscopy, images were also taken at random locations throughout the grid. Other experiments in this study were not subjected to randomization as the identity of the samples are predetermined during experiments; the experimental results would not be interpretable if these samples were randomized.
Blinding	When performing experiments, there were no samples that could be blinded as the identity of the samples are predetermined during experiments.

Reporting for specific materials, systems and methods

We require information from authors about some types of materials, experimental systems and methods used in many studies. Here, indicate whether each material, system or method listed is relevant to your study. If you are not sure if a list item applies to your research, read the appropriate section before selecting a response.

Materials & experimental systems

n/a	Involved in the study
☐	☒ Antibodies
☐	☒ Eukaryotic cell lines
☒	☐ Palaeontology and archaeology
☐	☒ Animals and other organisms
☒	☐ Clinical data
☒	☐ Dual use research of concern
☒	☐ Plants

Methods

n/a	Involved in the study
☐	☒ ChIP-seq
☐	☒ Flow cytometry
☒	☐ MRI-based neuroimaging

Antibodies

Antibodies used

Rabbit anti-HA C29F4 (Cell Signaling Technology, Cat#3724S), Mouse MBP Tag antibody(8G1) (Cell Signaling Technology, Cat#2396), PE anti-mouse CD4 Antibody (Biolegend, Cat#100408), APC/Cyanine7 anti-rat CD90/mouse CD90.1 (Thy-1.1) Antibody (Biolegend, Cat#202520), Brilliant Violet 421™ anti-mouse CD152 Antibody (Biolegend, Cat#106311), Pacific Blue™ anti-mouse CD25 Antibody (Biolegend, Cat#102022), Ultra-LEAF™ Purified anti-mouse CD3ε Antibody (Biolegend, Cat#100340), Ultra-LEAF™ Purified anti-mouse CD28 Antibody (Biolegend, Cat#102116), Anti-rabbit IgG HRP-linked Antibody (Cell Signaling Technology, Cat#7074)

Validation

All primary antibodies were validated previously by the manufacturer. Citations of studies using these antibodies and user ratings are provided on the manufacturer's websites:
Rabbit anti-HA C29F4 (https://www.cellsignal.com/products/primary-antibodies/ha-tag-c29f4-rabbit-mab/3724?cart-quantity=0&logged_in=0&shop-eu-id=mv6dcjq4frogokejieqbp7fcamip6pfp&N=0+4294956287&Nrpp=200&No=3000&fromPage=plp);
Mouse MBP Tag antibody (https://www.cellsignal.com/products/primary-antibodies/mbp-tag-8g1-mouse-mab/2396);
PE anti-mouse CD4 Antibody (https://www.biolegend.com/en-gb/productstab/pe-anti-mouse-cd4-antibody-250?GroupID=BLG4745);
APC/Cyanine7 anti-rat CD90/mouse CD90.1 (Thy-1.1) Antibody (https://www.biolegend.com/en-gb/search-results/apc-cyanine7-anti-rat-cd90-mouse-cd90-1-thy-1-1-antibody-5157?GroupID=BLG10566);
Brilliant Violet 421™ anti-mouse CD152 Antibody (https://www.biolegend.com/en-gb/search-results/brilliant-violet-421-anti-mouse-cd152-antibody-7322?GroupID=BLG10448);
Pacific Blue™ anti-mouse CD25 Antibody (https://www.biolegend.com/en-gb/products/pacific-blue-anti-mouse-cd25-antibody-3315?GroupID=BLG10428);
Ultra-LEAF™ Purified anti-mouse CD3ε Antibody (https://www.biolegend.com/fr-lu/cell-health/ultra-leaf-purified-anti-mouse-cd3epsilon-antibody-7722?GroupID=BLG6744);
Ultra-LEAF™ Purified anti-mouse CD28 Antibody (https://www.biolegend.com/fr-fr/products/ultra-leaf-purified-anti-mouse-cd28-antibody-7733?GroupID=BLG1565);
Anti-rabbit IgG HRP-linked Antibody (https://www.cellsignal.com/products/secondary-antibodies/anti-rabbit-igg-hrp-linked-antibody/7074).

Eukaryotic cell lines

Policy information about cell lines and Sex and Gender in Research

Cell line source(s)

The A549 parental cell line was a kind gift from Dr. Susan Weiss (U Penn) and was authenticated by her lab to be the same as ATCC CCL-185. HEK293T cells were purchased from ATCC (CRL-11268). EL4 cell line was a gift from Dr.Christophe Benoist lab (Harvard Medical School) to be the same as ATCC TIB-39.

Authentication

No form of authentication was used for these cell lines

Mycoplasma contamination

These cells were verified to be mycoplasma free by using the MycoAlert Mycoplasma Detection Kit (Lonza, Cat. No. LT07-318).

Commonly misidentified lines
(See ICLAC register)

No commonly misidentified lines were used in this study.

Animals and other research organisms

Policy information about studies involving animals; ARRIVE guidelines recommended for reporting animal research, and Sex and Gender in Research

Laboratory animals

C57BL/6N mice, sourced from Taconic Biosciences, were housed in an individually ventilated cage system at the specific-pathogen-free New Research Building facility of Harvard Medical School. The mice were maintained at a controlled environment with a temperature of 20-22°C, humidity ranging from 40-55%, and a 12-hour light-dark cycle. The spleens of 12~14 weeks old female C57BL/6 mice were isolated for the study.

Wild animals

The study did not involve wild animals.

Reporting on sex

To keep consistency, 12~14 weeks old female C57BL/6 mice were used for the study.

Field-collected samples	The study did not involve samples collected from the field.
Ethics oversight	Harvard Medical Area (HMA) Standing Committee on Animals

Note that full information on the approval of the study protocol must also be provided in the manuscript.

Plants

Seed stocks	N/A
Novel plant genotypes	N/A
Authentication	N/A

ChIP-seq

Data deposition

☒ Confirm that both raw and final processed data have been deposited in a public database such as GEO.

☒ Confirm that you have deposited or provided access to graph files (e.g. BED files) for the called peaks.

Data access links<br>*May remain private before publication.*	To review GEO accession GSE232754:<br>Go to https://www.ncbi.nlm.nih.gov/geo/query/acc.cgi?acc=GSE232754<br>Enter token qzinugqcdrunjqn into the box
Files in database submission	Input for naked DNA pulldown rep1<br>Input for naked DNA pulldown rep2<br>MBP-pulldown naked DNA rep1<br>MBP-pulldown naked DNA rep2<br>FoxP3-pulldown naked DNA rep1<br>FoxP3-pulldown naked DNA rep2<br><br>Input for nucleosome pulldown rep1<br>Input for nucleosome pulldown rep2<br>MBP-pulldown nucleosome rep1<br>MBP-pulldown nucleosome rep2<br>FoxP3-pulldown nucleosome rep1<br>FoxP3-pulldown nucleosome rep2<br><br>EV ChIPseq rep1<br>EV ChIPseq rep2<br>WT ChIPseq rep1<br>WT ChIPseq rep2<br>R356E ChIPseq rep1<br>R356E ChIPseq rep2<br>R396E ChIPseq rep1<br>R396E ChIPseq rep2<br>V408M ChIPseq rep1<br>V408M ChIPseq rep2<br>AAA ChIPseq rep1<br>AAA ChIPseq rep2
Genome browser session<br>(e.g. UCSC)	UCSC Mouse mm10

Methodology

Replicates	2 replicates
Sequencing depth	20M PE-150 paired-end reads
Antibodies	Mouse MBP Tag antibody(8G1) (Cell Signaling Technology, Cat#2396);<br>Rabbit anti-HA C29F4 (Cell Signaling Technology, Cat# 3724S)
Peak calling parameters	macs2 callpeak -c control.bam -t test.bam -p 0.01 -f BAM -g mm -n test_vs_control_0.01<br>macs2 callpeak -c control.bam -t test.bam -p 0.05 -f BAM -g mm -n test_vs_control_0.05
Data quality	First we do the quality QC test to ensure the sequencing quality using FastQC; Use Trimmomatic to remove adapters (LEADING:3 TRAILING:3 MINLEN:15); Use Bowtie2 to align reads to the genome, and the alignment rates are all over 89%.

	For FoxP3vsInput, 134,561 peaks are called at FDR 5%; For FoxP3vsMBP, 181,090 peaks are called at FDR 5%;
Software	Trimmomatic (version 0.36); Bowtie2 (version 2.3.4.3); Macs2 (version 2.1.1.20160309); DiffBind (version 4.3); Bedtools (version 2.30.0); Meme Fimo (version 5.3.3); Samtools (version 1.9); Deeptools (version 3.0.2)

Flow Cytometry

Plots

Confirm that:

☒ The axis labels state the marker and fluorochrome used (e.g. CD4-FITC).

☒ The axis scales are clearly visible. Include numbers along axes only for bottom left plot of group (a 'group' is an analysis of identical markers).

☒ All plots are contour plots with outliers or pseudocolor plots.

☒ A numerical value for number of cells or percentage (with statistics) is provided.

Methodology

Sample preparation	Naïve CD4+ T cells were isolated by negative selection from mouse spleens using the isolation kit (Miltenyi Biotec) according to the manufacturer's instructions. The purity was estimated to be >90% as measured by PE anti-CD4 (Biolegend) staining and FACS analysis. Naïve CD4+ T cells were then activated with anti-CD3 (Biolegend), anti-CD28 (Biolegend) and 50 U/mL of IL2 (Peprotech) in complete RPMI medium (10% FBS heat-inactivated, 2 mM L-Glutamine, 1 mM Sodium Pyruvate, 100 µM NEAA, 5 mM HEPES, 0.05 mM 2-ME). The activation state of T cells was confirmed with increased cell size and CD44 (BioLegend) expression by FACS. After 48 hours, cells were spin-infected with retrovirus using supernatant from HEK293T cells that were transfected with retroviral plasmids including MSCV-IRES-Thy1.1 expressing FoxP3. T cells were cultured in complete RPMI medium with 100 U/mL of IL2 for 2~3 days before harvesting and analysis.
Instrument	FACSCanto; SELF-FACSARIA II
Software	BD FACSDiva and FlowJo
Cell population abundance	The purity of the isolated CD+T cells was estimated to be >90% as measured by PE anti-CD4 (Biolegend) staining and FACS. analysis.
Gating strategy	When analyzing CD25 or CTLA4, a homogenous population were first gated on FSC-A/SSC-A, then singlets were gated on FSC-A/FSC-H, and then cells were plotted as CD4+ vs CD4- T cells via PE staining. Gates for FoxP3-transduced CD4+T cells were determined by comparing to mock transduced samples.

☒ Tick this box to confirm that a figure exemplifying the gating strategy is provided in the Supplementary Information.
